# No specific relationship between hypnotic suggestibility and the rubber hand illusion

**DOI:** 10.1038/s41467-022-28177-z

**Published:** 2022-01-28

**Authors:** H. Henrik Ehrsson, Aikaterini Fotopoulou, Dominika Radziun, Matthew R. Longo, Manos Tsakiris

**Affiliations:** 1 Department of Neuroscience, Karolinska Institutet, Stockholm, Sweden; 2 Clinical, Educational and Health Psychology Research Department, University College London, London, UK; 3 Department of Psychological Sciences, Birkbeck, University of London, London, UK; 4 The Warburg Institute, School of Advanced Study, University of London, London, UK; 5 Lab of Action & Body, Department of Psychology, Royal Holloway, University of London, Egham, Surrey UK; 6 Department of Behavioural and Cognitive Sciences, Faculty of Humanities, Education and Social Sciences, University of Luxembourg, Esch-sur-Alzette, Luxembourg

**Keywords:** Perception, Human behaviour

**arising from** Lush et al. *Nature Communications* 10.1038/s41467-020-18591-6 (2020)

In a recent study, Lush et al.^1^ claimed that they found substantial relationships between hypnotizability and experimental measures of the rubber hand illusion (RHI)^2^. The authors proposed that hypnotizable participants control their phenomenology to meet task expectations arising from the experimental paradigm. They further suggest that the RHI may or may not be entirely explained by hypnotic suggestions driven by task expectancies and therefore could reflect top-down control of perception, instead of multisensory mechanisms. However, in reanalyzing their data, we observe no significant relationships between hypnotic suggestibility and the RHI when quantified using a control condition in line with standard practice in the field and the authors’ preregistered hypothesis. Furthermore, we note that the relationships the authors describe are weak and observed for a visual hallucination control experience and in the control condition, indicating a general influence of hypnotizability on cognition, rather than sensations that specifically relate to the RHI. Overall, the results from our analyses and Lush original paper fit well with the view that the RHI is a perceptual illusion driven primarily by multisensory mechanisms.

During the RHI, participants experience a fake hand as their own^2^. In the classic version, synchronous versus asynchronous brushstrokes are applied to the rubber hand in full view of the participant and to the participant’s real hand, which is hidden from sight; the synchronous mode of stimulation elicits the illusion of sensing touch on the rubber hand and feeling that the fake hand is one’s own. Asynchronous stroking abolishes or significantly reduces the illusion, which is a commonly used control condition^2–4^. Although different theoretical models of the RHI have been proposed^5–7^, there is substantial agreement that the RHI is a perceptual illusion that arises from a combination of bottom-up and top-down processes involved in multisensory integration.

In contrast with this view, Lush and colleagues^1^ concluded that the RHI arises from hypnotic suggestibility at least partially, and they claimed that trait phenomenological control predicts the RHI. A total of 353 participants were tested on the RHI using the synchronous (illusion) and asynchronous (control) conditions. One-third of the participants were told that they would experience a stronger illusion in the synchronous conditions, another third were told that the effect would be stronger in the asynchronous condition, and the remaining participants were not given instructions. According to the authors’ predictions, these different suggestion instructions should influence expectations and the RHI, but this manipulation failed (see further below). The RHI was assessed using a standard questionnaire and a ‘proprioceptive drift test’ that assessed the change in hand position sensed toward the rubber hand. Individual differences in hypnotic suggestibility were quantified using the Sussex-Waterloo Scale of Hypnotizability (SWASH)^1^. Importantly, when contrasting the synchronous and asynchronous conditions in the group that received no suggestion instructions, in line with the preregistered hypothesis, no evidence for a relationship between the SWASH scale and the illusion ratings was observed, and only anecdotal evidence in favor of a relationship with proprioceptive drift was shown (non-significant two-tailed, *p* = 0.074)^1^. We reanalyzed the entire sample to maximize the power and observed non-significant relationships for both illusion ratings (Fig. 1a; *p* = 0.2187) and for proprioceptive drift (Fig. 1b; *p* = 0.3758). Moreover, a Bayesian analysis indicated that, for the illusion ratings, the null hypothesis was 5.68 times more likely than the alternative hypothesis (Fig. 1a); and when analyzing the illusion statement individually we observed similar convincing evidence against the preregistered hypothesis (Suppl Fig. 1A–C). Furthermore, when dividing the participants into four quartiles based on their SWASH scores, we observed that all four groups showed significant differences (*p* < 0.001) between the synchronous and asynchronous conditions on the illusion questions (Fig. 1c). Furthermore, all four groups affirmed the illusion experience in the synchronous condition in that their ratings were significantly greater than zero (*p* < 0.001; Fig. 1c). Thus, even the least suggestible participants showed clear evidence of experiencing the illusion.

**Fig. 1:**
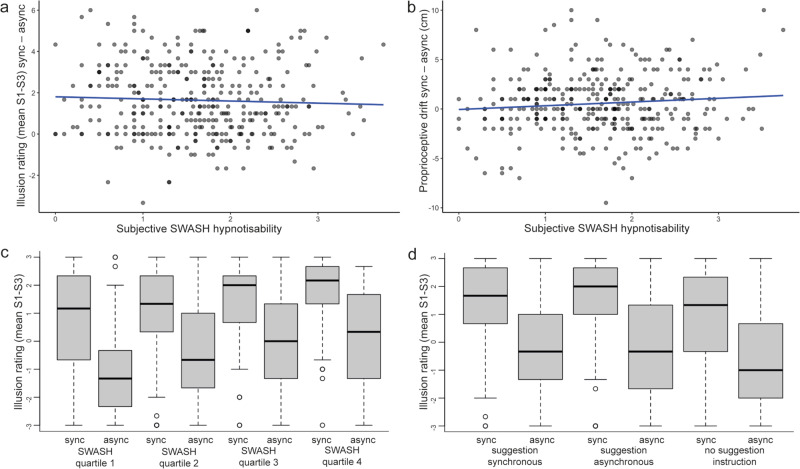
The rubber hand illusion (RHI) is resistant to hypnotic suggestibility. **a** No significant relationship between trait hypnotic suggestibility (SWASH) and illusion ratings (mean of questionnaire statements S1 to S3) when comparing the synchronous (illusion) and asynchronous (control) conditions (ϱ = −0.066, *p* = 0.219, 95% CI [−0.169, 0.039], BF_01_ = 5.685, *n* = 353). **b** Similarly, there was no significant relationship between SWASH and proprioceptive drift when contrasting the conditions (ϱ = 0.047, *p* = 0.376, 95% CI [−0.057, 0.151], BF_01_ = 1.657, *n* = 353). **c** When dividing the participants into four quartiles based on the trait of hypnotic suggestibility (SWASH quartiles 1–4), the illusion is significantly induced in all groups, including the least hypnotically suggestible subjects (quartile 1). The differences in illusion ratings between the synchronous and asynchronous conditions were significant in all four quartiles (1: *V* = 2575.5, *p* < 0.001, *n* = 88; 2: *V* = 2932, *p* < 0.001, *n* = 89; 3: *V* = 3198, *p* < 0.001, *n* = 92; 4: *V* = 2779.5, *p* < 0.001, *n* = 84), and the synchronous conditions were always rated significantly >0 (1: *V* = 2387.5, *p* < 0.001; 2: *V* = 3258.5, *p* < 0.001; 3: *V* = 3724, *p* < 0.001; 4: *V* = 3271.5, *p* < 0.001). Moreover, non-parametric one-way ANOVA (Kruskal–Wallis H test) on difference in illusion ratings between the synchronous and the asynchronous conditions in four quartiles was not significant (*χ*2 = 2.913, df = 3, *p* = 0.405; a direct comparison between quartile 1 and quartile 4 in terms of condition-specific differences was also non-significant: *W* = 3879.5, *p* = 0.574). **d** Explicit suggestions before the experiment commenced did not influence the illusion since the difference in illusion rating between the synchronous and asynchronous condition was not significantly different in the three groups that were informed that they would experience the strongest illusion in the synchronous condition (suggestion synchronous; *n* = 114), in the asynchronous condition (suggestion asynchronous; *n* = 115) or when receiving no information (no suggestion instruction; *n* = 124) (suggestion sync vs suggestion async: *W* = 6382, *p* = 0.73; suggestion sync vs no suggestion instruction: *W* = 7178, *p* = 0.836; suggestion async vs no suggestion instruction: *W* = 7422.5, *p* = 0.584). In all three groups, the illusion was significantly induced when contrasting the two conditions (*V* = 5438, *p* < 0.001; *V* = 4697, *p* < 0.001; *V* = 5169.5, *p* < 0.001). The data from Lush et al.^1^ are openly available and were analyzed with RStudio software, version 1.3.1056, and the BayesFactor software package, version 0.9.12–4.2. Non-parametric tests were used (Wilcoxon signed-rank test for paired comparisons, Mann–Whitney *U* test for independent group comparisons) since the questionnaire data were ordinal and not normally distributed; two-tailed tests were performed. For the Bayes factor analysis, the default Cauchy priors were used. Sync synchronous condition, Async asynchronous condition, SWASH Sussex-Waterloo Scale of Hypnotizability. The boxplots (in **c** and **d**) depict the data based on their median (thick black line) and quartiles (upper and lower ends of boxes). The vertical lines, i.e., the whiskers, indicate the minimum or maximum values within 1.5x the interquartile range above and below the upper and lower quartile. The circles denote outlier observations, the furthest being the minimum or maximum values in the data.

Following the unsuccessful attempt to manipulate RHI expectations by explicit suggestion and inconclusive results in preregistered analyses, Lush and colleagues used the whole sample to examine post hoc, exploratory correlations between the SWASH score and the two illusion measures when considering the synchronous condition in isolation. However, the hypnotic suggestibility trait accounted for only ~9% of the illusion ratings’ variability. A similar relationship was found for the asynchronous control condition (9%) and a control statement describing an ‘impossible’ experience of visual hallucinations (12%). Noteworthy, when subtracting the control statement ratings from the illusion ratings no positive relationship with SWASH was observed (Suppl Fig. 1D–F). Thus, hypnotizability seems to influence cognition and experience rather broadly, but it does not drive the perceptions that specifically relate to the illusion condition, i.e., the sensing of touches on the synchronously stroked fake hand and the condition-specific increase in the feeling of rubber hand ownership. Moreover, due to the nonspecificity of the post hoc findings, it cannot be excluded that they relate to different types of cognitive biases, including behavioral compliance or response bias, rather than ‘genuine’ phenomenological control effects.

The authors’ argument for leaving out the asynchronous control condition from the analysis was that including it is supposedly not motivated by the literature. In support of this, they cited the 20 most cited publications on the RHI using questionnaires or proprioceptive drift measures, respectively, resulting in a list of 30 studies in total (Supplementary information in Lush et al.^1^). However, and contrary to their claims, 28 of the 30 studies used the asynchronous control to conduct critical statistical comparisons with the synchronous condition. Moreover, their list: (1) referred to different illusion paradigms; (2) was small in comparison to the relevant RHI studies that operationalized the illusion effect as differences between conditions; and (3) was not supported by recent reviews on the RHI that emphasized the importance of using control conditions^4,6^. Thus, in our view, the previous literature was not reviewed correctly, and the results of the preregistered analysis concerning condition-specific relationships deserve more careful consideration.

A critical idea in phenomenological control theory is that synchronous and asynchronous conditions differ in demand characteristics and implicit suggestions. Therefore, these conditions trigger different phenomenological control, leading to the observed differences in the RHI measures. However, this hypothesis is not supported by the results, nor does it fit with the past literature. If differences in demand characteristics and implicit suggestions between the two conditions lead to different degrees of phenomenological control, as the authors theorized, this effect should be most pronounced in highly hypnotizable individuals. However, neither the findings of our analysis (Fig. 1a–c) nor the preregistered analysis^1^ support this prediction. Furthermore, if the condition-specific differences are related to demand characteristics, they should be affected by explicit suggestions. However, we observed no significant differences in RHI ratings among the three groups that received different suggestion instructions in our analysis (Fig. 1d). These observations do not support the authors’ theory but are well in line with the view that the RHI is a perceptual illusion, which should be resistant to suggestions, thoughts, and high-level conceptual knowledge.

A related concern is that when discussing prior literature, the authors emphasized the effect of demand characteristics in RHI paradigms and did not address in full the past literature that attempted to minimize and control it in various ways. For example, they did not consider previous studies that associated synchrony-asynchrony differences in the RHI to the temporal window of multisensory integration. Such studies systematically manipulated the degree of asynchrony and found that the illusion gradually diminished as visual and tactile stimuli became increasingly temporally incongruent, and the RHI was abolished for asynchronies >200–300 ms^8–11^. This precise temporal rule fits well with the temporal window of integration of multisensory cortical neurons^12^, causal inference theories of multisensory integration^7,11,13^, and the past body representation literature^2,3,5,7–11,14,15^. However, this precise temporal rule is not explained by phenomenological control theory because suggestions have been well controlled in studies using subtle experimental manipulations of asynchrony. Psychophysical approaches that better control cognitive bias have also been used in RHI studies with subtle asynchrony manipulations^11^.

In summary, the results from the study by Lush and colleagues^1^ provide an addition to the growing literature on individual differences in the RHI. However, they do not present evidence that fundamentally alters our understanding of the relationship between top-down control of experience and perceptual mechanisms.

## Reporting summary

Further information on research design is available in the Nature Research Reporting Summary linked to this article.

## Supplementary information

Supplementary Information

Reporting Summary

## Data Availability

The data is from Lush et al.^1^ and is publicly available at: https://osf.io/huwxd/. The source data underlying Fig. 1 and Supplementary Fig. 1 is provided as a Source Data file. Source data are provided with this paper.

## Source data

Source Data
