# Supplementary material for: No specific relationship between hypnotic suggestibility and the rubber hand illusion
Source: Nat Commun. 2022 Jan 28;13:564. doi: 10.1038/s41467-022-28177-z (PMC8799653; doi:10.1038/s41467-022-28177-z)
Supplement: Supplementary file 1 — Supplementary Information [file 41467_2022_28177_MOESM1_ESM.pdf]

Matters Arising: No specific relationship between hypnotic suggestibility and the rubber hand illusion

Ehrsson et al

Supplementary Figure.

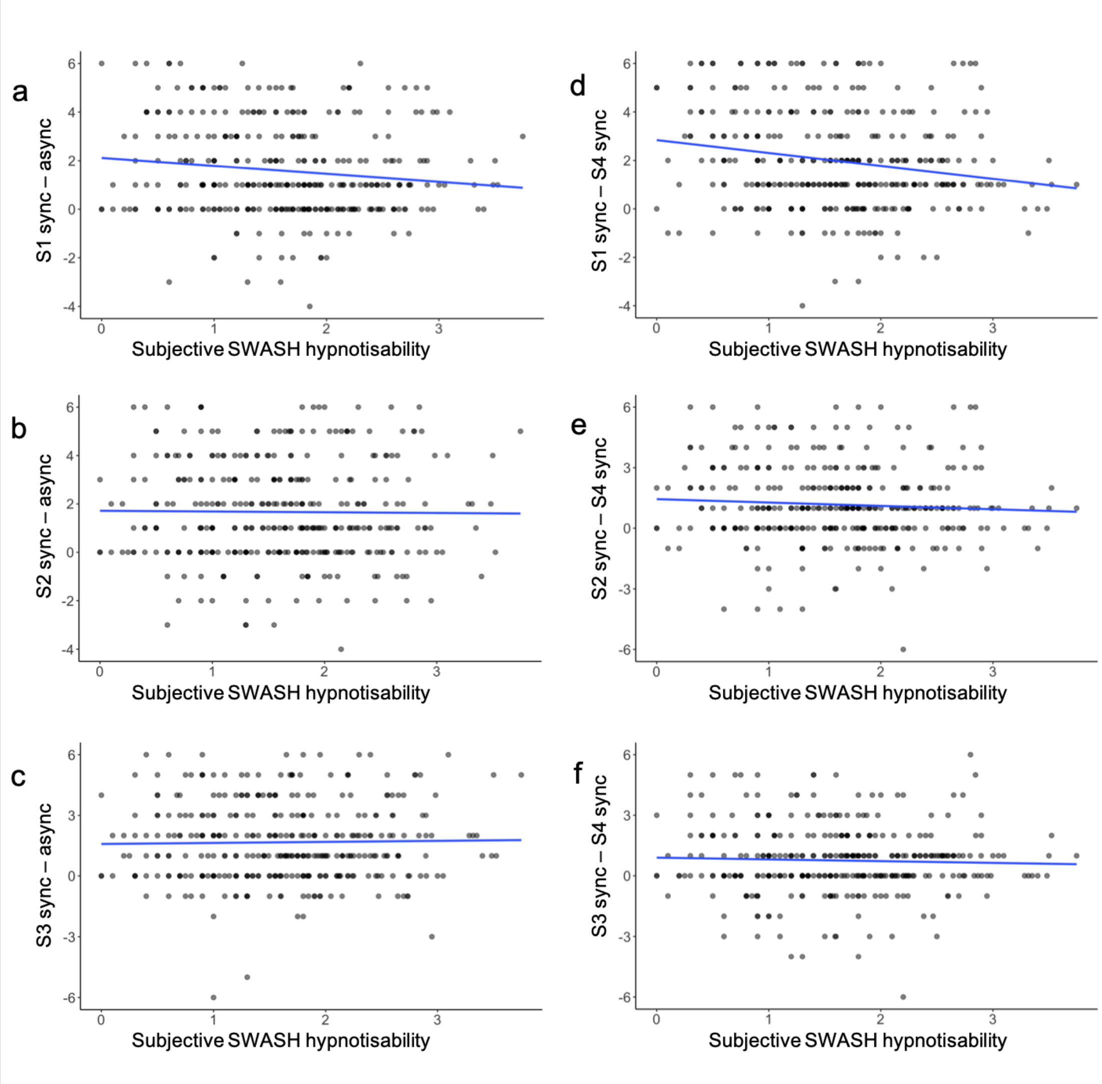

Supplementary Fig. 1. A. Significant *negative* relationship between trait hypnotic suggestibility (SWASH) and the ratings on the first illusion statement (S1), referring to sensing touch on the rubber hand when comparing the synchronous (illusion) and the asynchronous (control) conditions (ϱ = -0.137, p = 0.01, 95% CI [-0.238, -0.033], BF_{01} = 0.442654, n = 353). That is, a negative relationship directly *against* the authors' (one-tailed) pre-registered hypothesis. **B.** No significant relationship between trait hypnotic suggestibility (SWASH) and the ratings on the second illusion statement (S2), which relates to experiencing the touch one feels being caused by the touches one sees on the rubber hand, when comparing the synchronous (illusion) and the asynchronous (control) conditions (ϱ = -0.022, p = 0.686, 95% CI [-0.126, 0.083], BF_{01} = 7.88, n = 353). **C.** No significant relationship between trait hypnotic suggestibility (SWASH) and ratings on the third illusion statement (S3) that refers to the explicit feeling of ownership of the rubber hand when comparing the synchronous (illusion) and the asynchronous (control) conditions (ϱ = 0.003, p = 0.962, 95% CI [-0.102, 0.107], BF_{01} = 7.525, n = 353). **D.** Significant *negative* relationship between trait hypnotic suggestibility (SWASH) and the rating difference score when comparing the illusion statement S1 to control statement S4 (ϱ = -0.189, p < 0.001, 95% CI [-0.288, -0.086], BF_{01} = 0.009, n = 352); that is a relationship that goes *against* the predication of the phenomenological control hypothesis. **E.** No significant relationship between trait hypnotic suggestibility (SWASH) and the rating difference score when comparing the illusion

statement S2 to control statement S4 (ϱ = -0.063, p = 0.239, 95% CI [-0.166, 0.042], BF_{01} = 3.831, n = 352). **F.** No significant relationship between trait hypnotic suggestibility (SWASH) and rating difference score when comparing the illusion statement S3 to control statement S4 (ϱ = -0.013, p = 0.805, 95% CI [-0.118, 0.091], BF_{01} = 6.257, n = 352).
